# The structure and dynamics of Nano Particles encapsulated by the SDS monolayer collapse at the water/TCE interface

**DOI:** 10.1038/srep37386

**Published:** 2016-11-17

**Authors:** Wenxiong Shi

**Affiliations:** 1 School of Materials Science and Engineering, Nanyang Technological University, 639798, Singapore

## Abstract

The super-saturated surfactant monolayer collapses with the nanoparticles (NPs) at the water/trichloroethylene (TCE) interface are investigated using molecular dynamics (MD) simulations. The results show that sodium alkyl sulfate (SDS) monolayer collapse is initiated by buckling and followed primarily by budding and the bud encapsulating the NPs and oil molecules. The developed bud detaches from the monolayer into a water phase and forms the swollen micelle emulsion with NPs and oil molecules. We investigate the wavelength of the initial budding and the theoretical description of the budding process. The wavelength of the monolayer increases with bending modulus. The energy barrier of the budding can be easily overcome by thermal fluctuation energy, which indicates that budding process proceeds rapidly.

The gas/liquid interface is the traditional medium for the assembly of inorganic ions, biomacromolecules, and inorganic nanoparticles. Typically, the langmuir-blodgett (L-B) technique is used to prepare two-dimensional molecular assemblies^1^,^2^,^3^. For example, Rogach and coworkers reported that the CdSe and core/shell CdSe/ZnS nanocrystals self-assembled into large area periodic lateral structures at the gas/liquid interface^4^. However, the limitations of the self-assembly at the gas-liquid interface emerged, such as the low stabilization, and the 2D structure limitation. Therefore, the self-assembly of nano-material at the liquid/liquid interface is believed to have wide potential in natural and industrial application^5^. Today, more and more work focuses on fabricating the new nanoscale substances by self-assembly of unit molecules and nanomaterials at the liquid/liquid interface^5^. For instance, the researchers created CdSe ultra-thin crystal film^6^, which comprised by 10–50 nm Ag nanoparticles (NPs)^7^. Recently, Dai *et al.* have reported the first MD simulation of the *in situ* self-assembly of NPs and sodium alkyl sulfate (SDS) at a water/trichloroethylene (TCE) interface, highlighting the potential of using the liquid/liquid interface to produce novel nanomaterials^8^. Despite the self-assembly of nanoparticles at the liquid/liquid interface is receiving ever-increasing attention from both practical and theoretical points of view, the fundaments and principles for the NPs self-assembly are required further explored^8^.

Recently, the experimenters have done a great deal of work to study the NPs self-assembly on the liquid/liquid interface, the NPs encapsulation on the liquid/liquid interface, and the transportation of NPs across the interface^9^,^10^,^11^. The self-assembly of NPs can be controlled by the size of NPs and the types of ligands in the outer layer of NPs. Because of the different ligands, the NPs can be effectively distributed and assembled on the immiscible solvent interface^9^,^10^,^11^. Dai observed the structures of hydrogen modified diamond-like carbon NPs (HCP) aggregates self-assembled at water-oil interface experimentally^11^. In the following procedure of self-assembly, the ligands can be cross-linked to generate new types of capsules, well-distributed filter materials, nanoreactors, and sustained release material. Emrick found the gold NPs decorated by the pegylated ligand self-assemble into microcapsules on the Oil/Water interface of the microemulsion^12^. To increase the stability of the gold NPs-coated capsules, the small molecules were introduced into the oil phase for reaction with the chain-end hydroxyl groups of the pegylated ligand. This NPs self-assembly on the liquid/liquid interface method combines the advantage of the self-assembly on the liquid/liquid interface such as controllable, predictable spatial location, and the special NPs properties such as fluorescent, superconductive, magnetic. Such multi-functional nano-structured capsule which has the packaging and transport properties can be widely used at drug sustained release and delivery^13^. The NPs encapsulated by monolayer would obtain smaller nano-aggregates^14^. In order to encapsulate and immunoisolate the cells for treatment of cancer and other illnesses, researchers have worked for a long time to fabricate the capsules with pores between 5 and 20 nm^15^,^16^,^17^,^18^. The drawbacks of routine approaches are the too broad size distribution of nano-capsule pores, or may require laborious processing for one capsule at one time. Moreover, during the *ex-situ* filling procedure in appropriate solvents, only the substances sufficiently small to pass the pores can be inserted into the capsule^19^. Therefore, finding the time-saving one-step method to fabricate microcapsules which have the narrow size distribution and where the substances can be pre-packaged is significant. Edwards found that if salt was introduced into the water phase, the gold NPs capped with stimuli-responsive copolymers would be transferred spontaneously from the water phase to toluene phase across the interface^20^. The fraction of NPs that transferred across the interface depended on the chemical composition of the capping copolymers and the diameter of NPs. Most of the experiments were limited to study the equilibrium structures, not the dynamic self-assembly process, and could not provide molecularly detailed information of interfacial properties such as the interfacial thickness and so on^11^. The molecularly detailed information will help us to understand the principles of NPs self-assembly.

In our former simulation of TCE/SDS/Water ternary system, at the high surface coverage (28.4 SDS/Å^2^), where the interface tension becomes ultra-low, even negative, the interface is then unstable and the monolayer surface is rippled and collapses^21^. We choose SDS because it is not only one of the most widely used surfactants and has many applications in industry and science, but also plays an important role in a number of the emerging fields, such as packaging design of nanomaterials, and the sustained-release of drug molecules^22^,^23^. The collapse transition is initiated by the buckling of monolayers, is followed by budding and detachment of the nanoscale swollen micelle from the monolayer^21^. The whole process can be divided into five stages corresponding to the bud morphologies, namely, the bending monolayer, the cap-shaped bud, the tubular bud, the bud with a constricted neck, and the swollen micelles. The micellar microemulsion which is receiving ever-increasing attention from both practical and theoretical points of view produced when the blend is stabilized by the thermal fluctuations of its internal interface^24^,^25^,^26^,^27^,^28^,^29^. The TCE swollen micellar microstructure, corresponding to a micellar microemulsion, formed via monolayer collapse transition, has a great potential of ordering nanoparticle and drug delivery as a result of encapsulating nanoparticles into the core of micellar microemulsions and transferring them from one matrix phase to another one. The releasing of drug, nutrients, and nutraceutical with poor water solubility can be controlled by the NPs encapsulated delivery systems^30^.

The monolayer collapse and NPs encapsulation processes have the dynamic microstructure which is hardly studied in the bulk and constrained state, therefore the mechanism is still very obscure. With the aid of the increase in computational power, MD simulations are a valuable complement to the experiments by providing molecular details. Therefore, the molecular mechanic of monolayer collapse and the NPs encapsulation needed further exploration. In our simulation, the NPs are distributed around the SDS tail to investigate the encapsulation caused by the SDS monolayer collapse.

## Method

Our studied liquid/liquid quaternary system is comprised of water and TCE two bulk phases with surfactants and NPs dispersed at their interfaces. The GROMACS 3.3 simulation package^31^ and GROMOS96 force field^32^ are used for all our MD simulations. The molecular models, such as HCP, employed in this study are similar to the ones used by Dai *et al.*^8^,^11^, who demonstrated that they are reasonable in reproducing available experimental and simulation data. The SDS molecule is constructed as a hydrocarbon chain of 12 united carbon atoms attached to an SO_4_ head group with its atoms explicitly modeled. Partial charges on the sulfate head group were adopted from Bruce *et al.*^33^,^34^,^35^. The initial coordinates for the surfactant analogs and TCE were generated from the small-molecule topology generator PRODRG^36^. The modified hydrocarbon NPs (mean diameter of 10 Å) were truncated from a diamond-like lattice made of carbon atoms that bonded in non-planar hexagonal structure and, saturated with united CH, CH2, and CH3 atoms. The water was modeled using the single point charge (SPC) model^37^, with the bond lengths and angles held constant through the use of the SETTLE algorithm^15^. Bond lengths of surfactants and TCE were constrained using the SHAKE algorithm with a tolerance of 10^−4^ 16.

We started our simulations from the preassembled system consisting of two abutting thick slabs of water and TCE with SDS monolayer and NPs at the two water/TCE interfaces as our former work^21^. The procedure for preparing an initial configuration was similar to that employed to simulate the liquid/liquid interface of multicomponent systems^17^,^33^,^34^. The SDS monolayers were set up by first placing a certain number of SDS in all-trans configuration in such a way that all headgroups were constrained in a thin slab with X-Y dimensions commensurate with those water/TCE interfaces. The HCP NPs were set up close to the tail groups of the SDS monolayer. Then four monolayer slabs were inserted into the water/TCE system by shifting the positions of two bulk phases upwards and downwards respectively. The corresponding number of sodium ions was randomly placed in the interfacial region by replacing water molecules. Thus, water and TCE slabs were now separated by the SDS and NPs monolayer. It should be noted that at first, the all the molecules, water, SDS, and TCE molecules don’t overlap with each other. The SDS on the two interfaces does not interact with each other due to enough water and TCE between them. After the system was set up, the whole system was subjected to the steepest descent energy minimization with a cutoff of 10 Å for van der Waals and Coulomb forces, then to further density equilibration and thermalization by NP_N_AT MD runs at an external normal pressure of 1 bar (along the Z axis) at 300 K. The NP_N_AT ensemble is applied for the simulations at fixed cross-sectional area. In this ensemble, the number of molecules (N), X -Y box dimensions (L_x_ and L_y_), the normal pressure (P_N_), and the temperature (T) are fixed. As a result, the box size in the normal direction, L_z_, fluctuates, in keeping with the condition that its conjugate variable P_N_ is constrained. The temperature was maintained at 300 K using the Berendsen temperature coupling method, and Berendsen bath coupling scheme was used to keep a constant normal pressure of 1 bar^18^. The cutoff distance for short-range nonbonded interactions (van der Waals and real-space Coulomb) was chosen to be 12 Å and long-range electrostatic forces were computed using the Particle Mesh Ewald approach^38^,^39^. A time step of 2 fs was employed. All the simulations were at least 6 ns long.

In order to study the effect of cell length of simulation box, a set of initial structures with different number of SDS per monolayer of N_SDS_ = 675 and 900, and with different number of NPs per monolayer of N_NPs_ = 0, 0, 81 and 144 were created exactly in the same way as above, corresponding to monolayer coverage in the range 28.4 Å^2^ per SDS (where he monolayer collapse and form microemulsion in our former work^21^) in cells of L_x_ × L_y_ = 120 Å × 120 Å and L_x_ × L_y_ = 160 Å × 160 Å. Previous simulations of monolayer collapse show that the size of the simulated system limits both the modes of monolayer collapse and the variety of the bulk (three-dimensional) phase that could be formed^25^. As a result, four initial structures with and without NPs in different simulation cells were obtained.

The interfacial structures and properties were characterized using the utilities available in GROMACS as well as codes developed by us. The interfacial tension *γ* is defined as the difference of the normal, P_N_, and lateral, P_L_, pressures in the box:


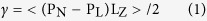


where L_z_ is the box normal size and P_L_ = (P_xx_ + P_yy_)/2. The factor (1/2) outside the bracket takes into account the fact that there are two interfaces in the system^40^.

## Results and Discussion

### NPs encapsulated by SDS monolayer collapse

**Figure 1:**
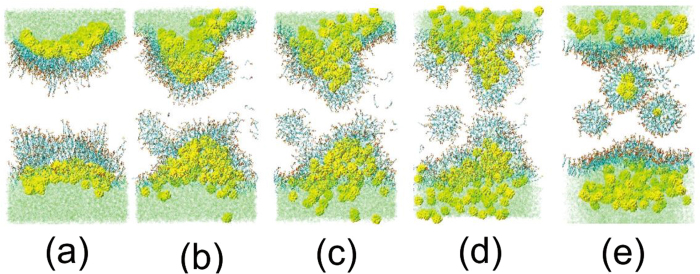
The snapshots of SDS monolayer collapse and encapsulate NPs in the L_x_ × L_y_ = 120 Å × 120 Å system at high interfacial coverage of 28.4 SDS/Å^2^. TCE is shown in green points; S atoms of SDS in yellow; O atoms of SDS in red; and SDS tails, in ochre bonds. NPs are shown in yellow beads. Water molecules (in the middle) are not shown. (**a**) 40 ps, (**b**) 400 ps, (**c**) 1120 ps, (**d**) 3000 ps, (**e**) 6000 ps.

The NPs encapsulated by SDS monolayer at water/TCE interface are discussed in this section. There are 2 buds appeared per monolayer and 3 swollen micelles with NPs encapsulated detached from the monolayer into the water phase, in the L_x_ × L_y_ = 120 Å × 120 Å system. An sample of NPs encapsulated by the collapse of SDS monolayer is presented in Fig. 1. For the considered range of 28.4 SDS/Å^2^, the monolayer is in the “liquid expanded (LE)” phase and unstable. At first, the monolayer increases its interfacial area by the development of curvature (Fig. 1a) and the NPs stay nearby the oil interface. With simulation time increasing, the budding deformations grow in amplitude and form cap-shaped bud (Fig. 1b), and the NPs enter the bud close to the end part of SDS’s tail. The bud proceeds into the water and further grows in amplitude and form tubular bud (Fig. 1c). Yang believes the tubular bud probably arises from the effect of monolayer thickness^41^. The tubular bud then forms a bud with the neck connected to the monolayer (Fig. 1d). The budding causes some of TCE molecules and NPs close to the interface to be encapsulated into the core of interior shell occupied by the SDS tails. However, SDS molecules are highly deformed at the connection of the bud to the monolayer. Then the neck shrinks its perimeter in order to minimize the energy of the bud-monolayer connection line, which eventually results in the pinching off of the swollen micelles from the monolayer (Fig. 1e). After the swollen micelles detach from the monolayer and diffuse into the bulk of the aqueous phase, the monolayer reforms a flat geometry and stabilizes at the interface. As shown in Table 1, the final interfacial tension after the collapse is very low, compared to the simulation data of 41.5 mNm^−1^ and the experimental result of 38.9 mNm^−1^ 8, and depends on the SDS monolayer surface coverage after the micelle detachment, which has a great agreement to our former work^21^. The final system consists of stable SDS monolayers at the water/TCE interface and swollen micelles. Corresponding to the bud morphologies, the whole collapse process which encapsulating the NPs can also be divided into the five stages, which is similar to the collapse process without NPs in our former work^21^.

### The structure of swollen micelle

**Figure 2:**
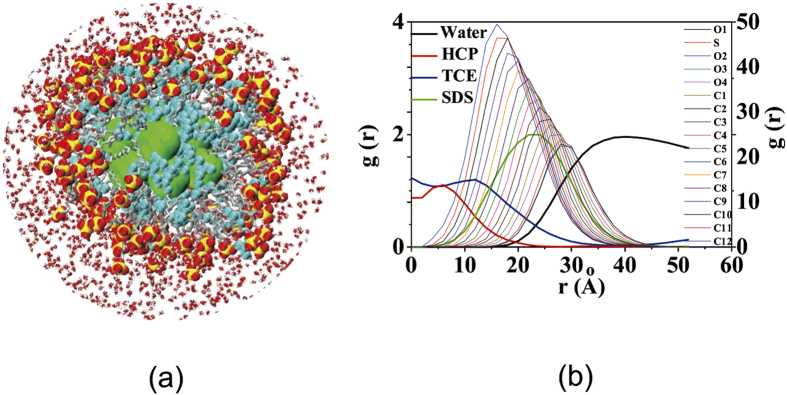
The structure of swollen micelle for the L_x_ × L_y_ = 120 Å × 120 Å system with NPs at 28.4 SDS/Å^2^ coverage, (**a**) profile of swollen micelle encapsulating TCE molecules and NPs, (**b**) The radial distribution function g(r) of the center of the swollen micelle and each group of the swollen micelle. The Water, TCE, SDS and NPs are normalized by space and density.

The interesting features of the swollen micelles structure can be depicted, as shown in Fig. 2. The swollen micelles contain NPs and TCE molecules (Fig. 2a) in the center of their core, which is surrounded by an outer core layer of SDS tails mixed with some TCE, and by the outer corona of SDS head-groups immersed into the external water phase. Nine NPs and 106 TCE molecules are encapsulated into swollen micelle as shown in Fig. 2a. The radius of SDS swollen micelles measured as the radial distribution function g(r) from the sulfur atoms of the head-group to the center of mass of TCE core is 45 Å, which is obviously larger than the reported values of pure SDS micellar radius of 22 Å 42. The radius of NPs core is 20 Å, and the radius of NPs and TCE core is 35 Å (Fig. 2b).

### The geometry of the budding

**Figure 3:**
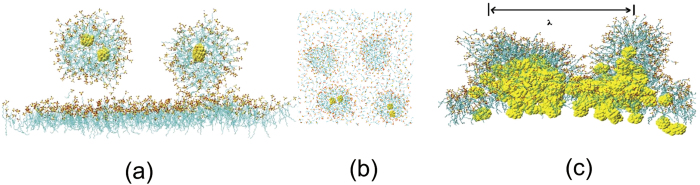
The final structure of one SDS monolayer collapse (**a**) side view, (**b**) top view, and the initial bud structure (**c**) in the L_x_ × L_y_ = 160 Å × 160 Å system with NPs. S atoms of SDS in yellow; O atoms of SDS in red; and SDS tails, in ochre bonds. NPs are shown in yellow beads.

In all the former simulations of L_x_ × L_y_ = 80 Å × 80 Å system, only one swollen micelle per monolayer was formed^21^. Our simulation boxes were enlarged to L_x_ × L_y_ = 120 Å × 120 Å and L_x_ × L_y_ = 160 Å × 160 Å. We found that in the L_x_ × L_y_ = 120 Å × 120 Å system, there are 2 buds appeared per monolayer and 3 swollen micelles detached from the monolayer into the water phase. More interestingly, there are 4 symmetrical buds appeared per monolayer and 6 swollen micelles detached from the monolayer into the water phase in the L_x_ × L_y_ = 160 Å × 160 Å system, as shown in Fig. 3a and b.

The similar phenomenon was found by Shinoda in coarse-grained simulation^43^. Because of the difference of the strength between dipalmitoyl phosphatidylcholine (DPPC) and polyethyleneglycol (PEG) monolayer, the persistence length, i.e., the correlation among the lateral lipids is different. The correlation length is quite large for a DPPC monolayer and very small for PEG monolayer. For DPPC, the buckling of the monolayer was found. However, only one small bud was detected, which were detached from the DPPC monolayer. For PEG, more than nine buds grew almost independently and detached from the monolayer.

We consider the wavelength λ of the bud before detaching from monolayer, defined as the distance from the center of the buds with a max amplitude, as shown in Fig. 3c 42. The wavelength of the L_x_ × L_y_ = 160 Å × 160 Å system is 70 Å. This clearly demonstrates that the budding has size constraints. The capillary wave of SDS monolayer before collapse has a certain correlation length and amplitude. The wavelength of the bud is approximate 70 Å. So that, only one bud appears at one monolayer in the L_x_ × L_y_ = 80 Å × 80 Å system^21^, and four buds appear at one monolayer in the L_x_ × L_y_ = 160 Å × 160 Å system. The budding of the monolayer is similar to the wrinkling of thin elastic sheets which follows simple scaling laws^44^. The wavelength of the wrinkles λ ∼ κ^−1/4^, where κ is the bending modulus. The wavelength is determined by the thicknesses and elastic properties of the film and the soft layer (subphase)^45^.


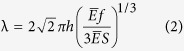


where the *h* is the thickness of the monolayer, and 
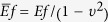
, *Ef* is the Young’s modulus of the monolayer, *v* is the Poisson’s ratio of the monolayer. The thickness of the SDS monolayer is about 15 Å, so that the 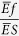 is about 3.48, which can be used to describe the wrinkle phenomenon^44^.

### Theoretical description of the budding

**Figure 4:**
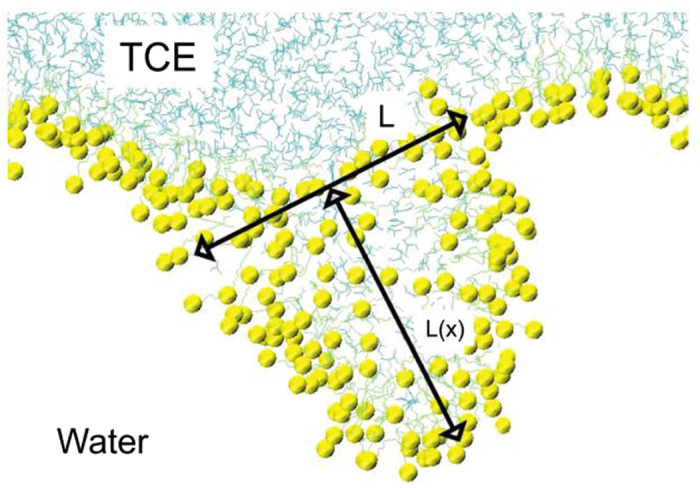
The side views of the bud in a monolayer. The length of the bud connection to the monolayer is *L*, and U_(0)_ is the value of the position-dependent height at the midpoint of the bud. TCE is shown in ochre lines; S atoms of SDS in yellow; and SDS tails in ochre lines.

Based on a mathematical analogy, Bruinsma^46^ propose a description of the reversible collapse for the formation of surfactant monolayer folds, which is similar to the Griffith Cracks formation of solid plates under stress. For a rectangular, self-adhering elastic sheet of thickness *h* with a two-dimensional (2D) shear modulus *G*, an area compressibility modulus *K*, a bending modulus *κ*, and self-adhesion energy per unit area *W*, The shape of a bud obey the following prediction:


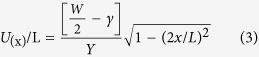


where the *U*_(x)_ is the height of the bud, L is the length of the bud connection to the monolayer, *γ* is the surface tension, and x is the coordinate along the bud connection, as shown in Fig. 4.

*U*_(0)_ is the max height of the bud. The bud should have a semielliptical shape according to the formula (3). Γ = U_(0)_/*L* is the ratio between the short and long axes of bud. *Y* is the 2D Young’s modulus, and


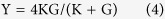


The area compressibility modulus of SDS at water-TCE interfaces in the LE phase obtained in our former work is 29.22 mNm^−1^ 21, which is close to the experimental results^47^.

The elastic force per unit length *γ+σ* pulling SDS out of the bud should equal the force per unit length *W/2* pulling SDS into the bud, where*σ* is the contribution to the elastic stress tensor introduced by the bud. For the long-tailed surfactant molecules monolayer, *W/2* can be identified as the free energy cost of creating a unit area of hydrocarbon surface, and is in the limited range of 25 ± 4 mNm^−1^ 46. We take the surface energy *W/2* of bud formation is approximate 22 mNm^−1^, just as DPPC and bulk alkanes^48^. The surface tension *γ* is approximate 0 mNm^−1^ near the collapse. Hence, *σ* must be a constant equal to *W/2 − γ*. The ratioΓ = U_(0_/*L* of SDS bud is approximate 1. Thus we can calculate the shear modulus *G* (6.8 mNm^−1^) using the formula (2), which is close to the experimental results 4 mN/m^49^. we also can obtain the 2D Young’s modulus *Y* (22 mNm^−1^) from the formula (4). All the modulus parameters were summarized in Table 2.

Similar to the critical crack size of solid plates, the critical bud length *L*_*cr*_ (above which the bud will grow, otherwise, the bud will shrink), is given by^46^.


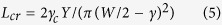


where *γc* is the line tension at the bilayer-monolayer connection. The *γ*_c_ can be estimated by using the following formula^48^.


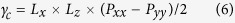


Then the *γ*_c_ is 12 pN in our SDS system according formula (5). The *γ*_c_ of the DPPC/POPG 4:1 monolayer is 48 pN and the *γ*_c_ of the softer DPPC/POPG 1:1 monolayer is 20 pN. Therefore the *γ*_c_ of SDS monolayer, with lower bending modulus, is smaller than the two lipid mixed monolayer (DPPC/POPG), which is reasonable^48^. Meanwhile, The energy barrier for budding can be obtained according to^46^.


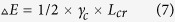


Using the calculated values, we obtain *L*_*cr*_ = 3.5 Å, and Δ*E* = 0.5*K*_*B*_*T*. The energy barriers can be easily overcome by thermal energy, which indicates that budding proceeds spontaneously and rapidly.

## Conclusions

In this work, the super-saturated SDS monolayer collapses with the NPs at the water/TCE interface are investigated using MD simulations. The results show that SDS monolayer collapse is initiated by buckling and the formation of nanometer-scale swollen micelles. This process is a potential mode of 3D relaxation of the monolayer at the water/TCE interface. The SDS monolayer collapse transition is followed primarily by budding and the bud encapsulates the NPs and oil molecules. The developed bud detached from the monolayer into a water phase and formed the swollen micelle emulsion with NPs and oil molecules. We investigate the wavelength of the initial budding and the theoretical description of the budding process. The wavelength of the monolayer increases with bending modulus^44^. The wavelength of the SDS monolayer is about 70 Å. The energy barrier of the budding can be easily overcome by thermal fluctuation energy, which indicates that budding proceeds spontaneously and rapidly. Therefore, this work moves a step forward to precisely predict the wrinkle phenomenon of super-saturated surfactant monolayer collapses with the nanoparticles (NPs) at the water/TCE interface.

## Additional Information

**How to cite this article**: Shi, W. The structure and dynamics of Nano Particles encapsulated by the SDS monolayer collapse at the water/TCE interface. *Sci. Rep.* **6**, 37386; doi: 10.1038/srep37386 (2016).

**Publisher’s note:** Springer Nature remains neutral with regard to jurisdictional claims in published maps and institutional affiliations.

## Figures and Tables

**Table 1:** The composition of the simulation systems.

System	NP (particle)	SDS (molecule)	TCE (molecule)	Water (molecule)	*γ* (mNm^−1^)
A1	0	1012	6056	46876	3.48
A2	162	1012	6056	46876	1.65
B1	0	1800	10450	83141	1.85
B2	288	1800	10450	83141	3.15

**Table 2:** Parameters of the simulation systems.

System	*K* (mNm^−1^)	*G* (mNm^−1^)	*Y* (mNm^−1^)	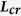 (Å)	*γ*_c_ (pN)	*γ* (mNm^−1^)	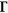	*W* (mNm^−1^)	Δ*E (K*_*B*_*T*)
A2	29.22	6.8	22	3.5	12	0	1	44	0.5
